# Cancer genomics just got personal

**DOI:** 10.1186/s13059-014-0464-5

**Published:** 2014-10-01

**Authors:** Rafal T Marszalek

**Affiliations:** Genome Biology, BioMed Central, 236 Gray’s Inn Road, London, WC1X 8HB UK

It was one Saturday morning in April when, after a 24-hour journey across half of the world, and a very early morning start to the 2014 AACR annual meeting marathon, I was sitting in a dark room in the San Diego Convention Center listening to Kornelia Polyak’s talk about the evolution of breast cancer. Polyak, an expert in breast cancer genomics, was reporting on some recent results from her group. Then, she made a statement that for many cancer researchers will perhaps seem obvious, but that was not yet obvious to me. Polyak noted that it is not uncommon that when several different clones of cancer are present in a patient, the clone that metastasizes is not the most aggressive one.

This does of course make a lot of sense: the clone that metastasizes doesn’t need to be aggressive at all. Rather, it needs to be resistant to therapy. And so it is quite logical that even if other, often more dominant and larger clones are destroyed by the treatment, the ones that were until then suppressed suddenly gain breathing space to grow and migrate. The idea resonated with me a lot, as it seemed to cover so many concepts and threads that we were already seeing in the submissions for the special issue: cancer heterogeneity, clonal diversity, therapy response and resistance, and progression of the disease. In one sentence Polyak unknowingly summarized the issue of *Genome Biology* that we now bring you.

## The cancer genomics explosion

When The Cancer Genome Atlas consortium kicked off nearly 10 years ago, the initial goal was technically challenging, but from today’s perspective seems rather modest: to characterize three types of human cancer. Five years ago it expanded into the next phase, which would focus on the analysis of around 20 more tumor types. By now the consortium has sequenced and analyzed the data from over 10,000 samples, characterizing over 30 tumor types [1].

Another consortium, the International Cancer Genomics Consortium, has also made its mark on cancer genomics: its goal is characterization of around 50 cancer types, and sequencing of over 25,000 samples. The work of both consortia led to a deluge of many types of ‘omic’ data being available to researchers all over the world, as well as to a series of high profile publications describing mutational landscapes in major cancer types.

These consortia did a tremendous job in providing the community with an incredible resource, but what is becoming increasingly clear, is that these analyses represent an average image of a given cancer: a moment frozen in time, which often doesn’t reflect the immense heterogeneity of the tumors between patients, nor the diversity seen within patients, nor the changes commonly observed as the cancer evolves and progresses. To address these issues, the next phase in cancer genomics research must involve expansion in a number of new directions.

First, we need to study cancer at different genomic levels, and analyse the data in an integrative way that can produce synergistic results (this, to a large extent, is already happening within the consortia frameworks). That, hopefully, will give us a better understanding of the cancer heterogeneity underlying, for instance, differences in therapy response. Second, we should look at cancers at different time points during their development: to get a better view of the mechanisms that lead to cancer progression; to better comprehend what happens when a selective pressure, in the form of therapy, is applied to the tumor; and to find new ways of fighting cancers that became resistant to treatment. Finally, we should take a closer look at individual patients. We should study their primary tumors alongside the metastatic lesions; and we should study their tumors in greater depth, to get a better view of rare events in their genomes that, while not necessarily easy to find, may just be the ones responsible for the disease outcome.

## Less is more

Looking in detail at individual patients to get a better picture of their personal ‘mutational (and other) landscapes’, trying to identify and characterise rare disease subtypes, working with samples for which no appropriate reference is available – all these themes underlying the articles of this special issue are combined in one general concept of personalized medicine. And it does indeed seem that the emphasis in cancer genomics is more and more on individuals [2].

This month we publish two elegant examples of N-of-1 studies, which demonstrate the power of thorough analysis of one individual. Hanlee Ji and colleagues in one of these studies [3], and Charles Swanton and colleagues in the other [4], study single cancer patients with hereditary gastric cancer and kidney cancer, respectively, and provide insights into the evolution of these cancer types. While Ji and colleagues show how such studies can be additionally informed by a clever choice of models for mechanistic studies, Swanton and colleagues focus on demonstrating how cancer development follows basic evolutionary principles.

A wonderful addition on this topic is an Opinion from Obi Griffith, Andrew Su and colleagues [5] who discuss a non-trivial issue of how to interpret the data from these N-of-1 studies, of which we are bound to see more and more. In their article they raise an interesting issue of personal investment: while that of the patient is obvious, we have to realize that personal investment of the researcher is also needed for us to be able to make the most of the data that we are so keen to produce, but which is getting increasingly harder to navigate and interpret.

## More is more

The rise of N-of-1 studies does not mean that large cohort sequencing is dead. What we already see is the expansion of projects dedicated to characterization of different cancer types. Now that several major, common cancer types have been analyzed, the aim will be to improve the picture that we have of them, for example to get a clearer view of how their subtypes differ. Our understanding of this basic heterogeneity is far from good, and this becomes clear when we realize how unrefined are common classifications of some of the most frequent cancer types, as demonstrated by Carlos Caldas, Samuel Aparicio and colleagues on the example of breast cancer [6,7]. For other cancers, our view of the different subtypes that exist is not even yet complete – Colin Collins and colleagues show, through the transcriptomic analysis of prostate cancer, that new, rare, and dangerous subtypes can still be identified [8].

We also have a lot to learn from the analysis of other data for cancers that are seemingly well-characterized. In the study from Christopher Maher and colleagues [9], a search through lung cancer transcriptomes reveals thousands of novel long intergenic non-coding RNAs, which are dysregulated in this cancer and affect its growth. Two other studies turn from gene expression to focus more on DNA methylation: a breast cancer study from Vessela Kristensen and colleagues [10] and a Wilms tumor study from Katherine Pritchard-Jones, Stephan Beck and colleagues [11] both reinforce the concept that aberrant methylation may give rise to new biomarkers of cancer progression. The idea of how this variety and abundance of data can be better used is further explored in an Opinion on integrative analyses by Anne-Lise Børresen-Dale and colleagues [12].

## Give me a lever, give me a tool

We may be keen to jump on the problem of cancer heterogeneity at once. With sequencing getting ever cheaper [13] and new technologies waiting for us around every corner [14], it may seem that addressing this challenge has never been easier. But we would be nowhere without the right analytical tools – and the wave of methods for the analysis of cancer evolution and phylogenies published recently [15-17] only goes to show that the need for a new toolkit is huge.

Therefore it should be no surprise that in this special issue we found a place for several excellent Method articles: Gabor Marth and colleagues describe SubcloneSeeker (the clue is in the name!) [18] and Francesca Demichelis and colleagues give us CLONET [19], both of which are designed for the analysis of clonality. Shirley Liu and colleagues [20] describe a tool for the identification of differential methylation in single tumor samples. Interesting and innovative approaches to cancer evolution studies are also presented in the Research articles from Olivier Elemento and colleagues [21] who look at lymphoma development through the prism of VDJ-region sequencing, and from Francesca Ciccarelli and colleagues [22] who study colorectal cancer by the means of X chromosome sequencing.

Another engaging read is an Opinion article on competition-based evaluation of bioinformatic tools [23], in which Gustavo Stolovitzky, Andrea Califano and colleagues discuss the benefits, and the pitfalls of these community efforts, which are being more and more often used to find the most suitable and imaginative tools to deal with complex bioinformatic problems.

## Without further ado

And so, without further ado, we invite you to browse through these, and other, articles included in this special collection on the genomics of cancer progression and heterogeneity [24]. While we realize that with this issue we barely scratched the surface of this area of research, every day we are reminded with new studies being published how much is being done and learned, and how much to do and to learn there still is.

## Acknowledgements

We would like to thank the authors of all manuscripts submitted for consideration in the issue; over 100 anonymous reviewers who helped to guide the manuscripts through the peer-review process; and the authors of all non-research content contributed to the issue.

Finally, we would like to express our deepest gratitude to two fantastic Guest Editors, Professor Elaine R Mardis and Professor Samuel Aparicio. We wish to thank them for their help in shaping the issue, for their invaluable advice and continued support.
